# Panalyze: automated virus pangenome variation graph construction, analysis and annotation

**DOI:** 10.1093/bioadv/vbag071

**Published:** 2026-03-10

**Authors:** Chandana Tennakoon, Thibaut Freville, Tim Downing

**Affiliations:** Pirbright Institute, Surrey, GU24 0NF, UK; Pirbright Institute, Surrey, GU24 0NF, UK; Pirbright Institute, Surrey, GU24 0NF, UK

## Abstract

**Motivation:**

Constructing and studying pangenome variation graphs (PVGs) supports new insights into viral genomic diversity. This is because such pangenomes are less prone to reference bias, which affects mutation detection. Interpreting the information arising from this is challenging, so automating these processes to allow exploratory investigations for PVG optimisation is essential. Moreover, existing methods do not scale well to the smaller virus genome sizes and to facilitate analysis in laptop environments. To address this, we developed an easily deployable pipeline to facilitate the rapid creation of virus PVGs that applies a broad range of analyses to these PVGs.

**Results:**

We present Panalyze, a computationally scalable virus PVG construction, analysis and annotation tool implemented in NextFlow and containerised in Docker. Panalyze uses NextFlow to efficiently complete tasks across multiple compute nodes and in diverse computing environments. Panalyze can also operate on a single thread on a standard laptop, and analyse sequence lengths of any size. We illustrate how Panalyze works and the valuable outputs it can generate using a range of common viral pathogens.

**Availability and implementation:**

Panalyze is released under a MIT open-source license, available on GitHub with documentation accessible at https://github.com/downingtim/Panalyze/.

## 1 Introduction

Pangenome variation graphs (PVGs) offer new insights into genome evolution over short and long scales. A PVG is a sequence graph whose nodes represent sequences and whose edges represent connections observed in data (Hein 1989) such that a genome can be decoded by traversing a series of paths (Garrison *et al.* 2018). PVGs combat reference allele bias by allowing reads to map to the most similar combination of paths in a PVG, instead of a single haplotype (Paten *et al.* 2018). Reference bias is not obviated by using a consensus reference sequence or by using a multi-sample reference (Chen *et al.* 2021, Chen *et al.* 2024). This affects the chance of a read mapping to a location, impacting mutation detection, phylogenetic analyses, and associated inferences in pathogens (Valiente-Mullor *et al.* 2021) particularly at variable regions that are often strongly associated with phenotypic changes (Haga *et al.* 2024). This results in unaligned reads, missed mutations, poorer inferences, and less precise evolutionary models (Baaijens *et al.* 2017, Hickey *et al.* 2020). Novel pathogen isolates genetically distinct from known references will be characterised less accurately (Boehm *et al.* 2021). In viruses, although genome assembly can be effective, extreme AT/GC content, host/vector contamination, short output contig lengths, and repetitiveness all contribute to incomplete and biased genomes (Alser *et al.* 2021, Lischer and Shimizu 2017). Moreover, contigs often need to be aligned to existing databases either as sequences or k-mers, or an existing unavoidably-biased scaffold may be used as a reference (Ondov *et al.* 2016, Bradley *et al.* 2019).

Only two studies have been published on viral PVGs. PVGs helped recover >8% of sequence that failed to align to known linear reference genomes (Duchen *et al.* 2024), and identify 27% more SNPs (Wright *et al.* 2025). A PVG can efficiently represent the diversity of multiple viral strains and so act as a better reference structure than a single linear reference (Downing 2026). This means reads can map to different parts of the haplotypes represented in the PVG, which reduces the effect of known mutations and improves mutation discovery (Paten *et al.* 2017, Rakocevic *et al.* 2019). This circumvents the issue of reference genetic distance, improves alignment quality, and may permit more accurate genetic reconstruction of the sample collection (Moshiri *et al.* 2022, Guarracino *et al.* 2023). Thus, there is a need for PVG creation and analysis to explore parameter spaces and optimise processes for viral PVGs.

There are many tools available for PVG creation and analysis. Most of these are developed with eukaryotic genome sizes in mind. These may require minimum sequence lengths larger than a typical virus genome length. There is a class of k-mer based reference-free methods that are fast when analyzing large genomes. However, with smaller sizes of virus genomes, we can efficiently use graph-based methods that provide richer structural representation. Moreover, many of these tools are hard to install due to complex installation procedures, assume numerous dependencies, and have steps requiring root access. Thus, it would be beneficial to have a curated set of pre-installed tools that can be easily deployed to automate PVG construction, analysis and annotation for small genome sizes. An existing tool, nf-core/pangenome (Heumos *et al.* 2024) achieves this for larger eukaryotic genome sizes, but does not work for genomes <5 Kb long, has limited analysis options, and requires a minimum number of computer server threads. Moreover, the smaller genome sizes of viruses means that their PVG analysis is possible on a laptop. Pandora is another alternative for bacterial pangenome graph construction and read mapping that does work on viral data, but lacks capacity to analyse PVGs (Colquhoun *et al.* 2021). Panalyze uses PGGB because of its versatility in downstream analyses, ability to produce variation graphs agnostic to genome type, and lack of licensing restrictions.

To address these issues, we present a scalable and rapid virus PVG construction, analysis and annotation tool operating in NextFlow v24.10.3 (Di Tommaso *et al.* 2017) and containerised in Docker (Merkel 2014) called Panalyze. It wraps a series of tools to allow for automated sequence retrieval, PVG creation, phylogenetic analysis, PVG visualisation using multiple methods, total PVG size calculation, PVG feature summarisation using different tools, PVG openness analysis, PVG core size estimation, VCF generation, BUSCO gene detection, PVG community detection and PVG annotation. Panalyze focuses on constructing PVGs for smaller (viral) genomes and on supporting the interpretation and analysis of PVGs. Panalyze can use cluster nodes efficiently in a scalable manner, or can be run on a single thread on a laptop or using many threads on a computer server. It also resolves the issue of complex installations and dependencies using Docker. We illustrate how Panalyze can work effectively for segmented, DNA and RNA viruses.

## 2 Methods

### 2.1 Pipeline overview

**Figure 1:**
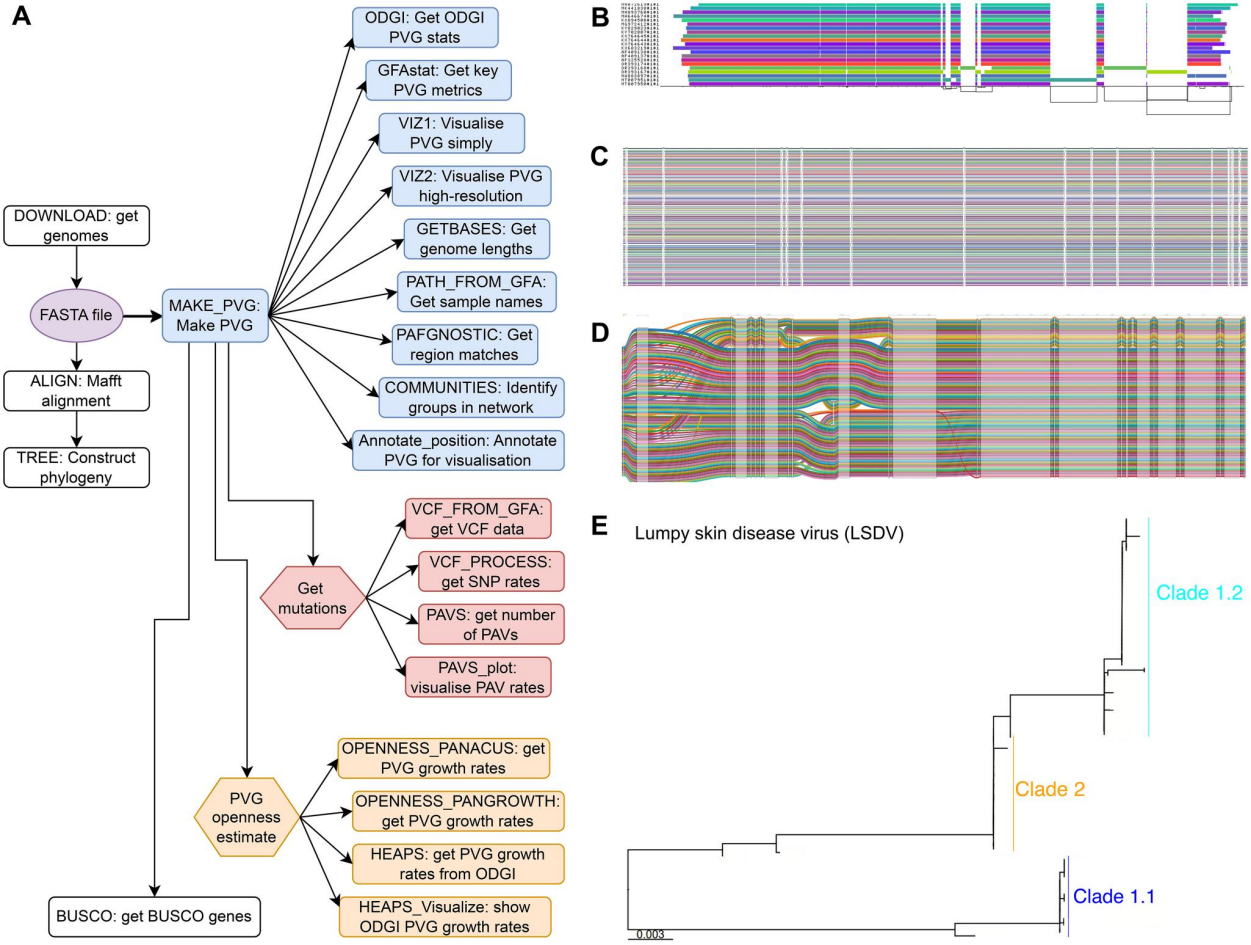
Panalyze’s workflow and outputs. (A) Optional modules are coloured white, PVG analysis ones are in blue, mutation analysis ones are red, and openness modules are in orange. Users can either retrieve sequences of interest via the DOWNLOAD process, or curate a sample set and start the pipeline at ‘FASTA file’ (purple oval). MAKE_PVG (blue) will take the FASTA to make the PVG, before running analyses (blue), including: getting PVG metrics with the ODGI and GFAstat processes, visualisations with VIZ1 and VIZ2, genome lengths with GETBASES, creating PAFs with PAFGNOSTIC, community detection with COMMUNITIES and annotation. Other processes are on mutation detection (red): determining VCFs with VCF_FROM_GFA, VCF_PROCESS to infer mutation rates, getting PAVS with PAVS, and visualising the PAVs with PAVS_plot. Users can implement openness analyses (orange) using Panacus, Pangrowth and ODGI, HEAPS and HEAPS_Vizualize. Users can (optionally) identify Busco genes with BUSCO. (B) A region at 135–140 Kb from a 121-sample LSDV PVG visualised using ODGI viz, where each genome shown is a coloured bar and the PVG topology is shown as the genomic coordinates the x-axis (black lines). (C) 136–138 Kb of the same PVG visualised with VG viz. (D) A zoom-in from the same region using SequenceTubeMap (Beyer *et al*. 2019), showing one minor (22 samples from LSDV Clade 1) and one major (99 samples from LSDV Clades 1.2 and 2) haplotype. (E) A phylogeny of this region showing the LSDV clades and their differentiation between these two LSDV haplotypes.

Panalyze integrates selected tools based on their reliability, effectiveness, and compatibility with downstream analysis; several alternatives were excluded due to poor maintenance or limited functionality. Panalyze has two entry points: a pre-existing FASTA file, or a search query from the user (Fig. 1A). The latter seeks all complete genomes or genomic sequences matching the query in the Nucleotide database (Sayers *et al.* 2021). The sample names are formatted to adhere to the PanSN-spec system (https://github.com/pangenome/PanSN-spec). The sequences are aligned with Mafft v7.453 (Katoh and Standley 2014) using automatic optimisation and default parameters. Evolutionary relationships are reconstructed using RAxML-NG (Randomised Axelerated Maximum Likelihood) v1.2.0 (Kozlov *et al.* 2019) with a GTR (general time reversible) model and gamma substitution rate heterogeneity. Other models can be selected with modeltest-ng (Darriba *et al.* 2020). Phylogenies are mid-pointed rooted and visualised using R v4.3.2 (R Core Team 2025) packages ape v5.7–1 (Paradis and Schliep 2019), ggtree v3.8.2 (Yu *et al.* 2017), phangorn v2.11.1 (Schliep 2011), Rcpp v1.0.11 (Eddelbuettel and Balamuta 2018), RcppArmadillo v0.12.6.6.0 (Eddelbuettel and Sanderson 2014), phytools v2.0–3 (Revll 2024), and treeio v1.24.3 (Wang *et al.* 2020).

Panalyze creates PVGs using the pangenome graph builder (PGGB) v0.4.0 pipeline (Garrison *et al.* 2024), which initiates all-to-all alignment with WFMASH v0.9.1 (Guarracino *et al.* 2021) subject to a threshold of 90% identity per Kb (Fig. 1B). If this fails to generate a valid PVG due to high similarity among the genomes, this is re-attempted with the identify threshold set to 50% and a k-mer mapping size altered to 11. PGGB keeps all paths and was used to reconstruct the input haplotypes directly. PGGB creates the PVG using SEQWISH v0.7.5–5 (Garrison and Guarracino 2023) using a k-mer of 19 bp and a window size of 27 bp, and then sorts and orders it to produce a progressively linearised PVG based on partial order alignment with SMOOTHXG v0.6.4. This also removes erroneous redundant nodes (Garrison and Guarracino 2023).

To allow analysis of a region <1.5 Kb in size, we pad the sequences with nucleotides to a minimum length of 1.5 Kb. Once a PVG is built, we break the PVG into single-nucleotide nodes, retaining the original topology. Then we remove the nodes belonging to the padded sequences. Finally, we collapse this pruned PVG so that consecutive unitigs (where present) are collapsed into contigs. When the mash algorithm is called with such a genome, the padding is entirely composed of N bases, which are ignored when detecting k-mers. The outputs of these programs are post-processed to reflect the original sequence.

Multiqc v1.14 (Ewels *et al.* 2016) collates PGGB metrics. The PVGs are generated in Graphical Fragment Assembly (GFA) format version 1. Panalyze does not use PVG pruning, chunking or lacing because virus PVGs are small. These PVGs are converted with the convert function in variation graph (VG) v1.43.0 (Garrison *et al.* 2018), indexed with VG’s autoindex function. The corresponding FASTA file is indexed with SAMtools (Li *et al.* 2009).

Next, a range of PVG analyses are applied. GFASTATS v1.3.6 (Formenti *et al.* 2022), Bandage (Wick *et al.* 2015), and ODGI (optimized dynamic genome/graph implementation) v0.8.3 (Guarracino *et al.* 2022) produce PVG summary statistics. Visualisations are created with ODGI (Fig. 1) and PGGB (Garrison *et al.* 2023). The number of bases in the PVG comes from ODGI. A VCF is produced by GFAUTIL v0.3.2 (Fischer 2021). The relative differences between the sequences’ coordinate systems is computed by GFAUTIL and visualised with ggplot2 v2_3.4.4 (Wickham 2016). PVG openness is computed firstly by Panacus v0.2.3 (Parmigiani *et al.* 2024a) using the nodes in the PVG with default coverage thresholds that included all segments, secondly with k-mers using Pangrowth (Parmigiani *et al.* 2024b), which also estimates the shared PVG size, and thirdly with sequences extracted from PVG nodes using ODGI’s heaps function using 1000 simulations. Three methods are used to ensure consistency of openness esti-mates. Pangrowth and Panacus provide faster sequence-based and k-mer-based estimates, while ODGI heaps offers unique insights by modelling openness as a function of sample size, enabling de-tection of inconsistencies and identifying the shared core PVG.

These PVG community data are parsed, visualised, and the pairwise alignment format (PAF) files are generated for input to PAFGNOSTIC to numerically summarise PVG properties. A tabular PVG annotation file is generated using Prokka (Seemann 2014) for visualisation with Bandage (Wick *et al.* 2015). Presence-absence variation (PAV) can be determined by ODGI and visualised, but should only be used for smaller or less diverse datasets due to the compute time required. Optional analyses include determining gene presence-absence via BUSCO (Manni *et al.* 2021).

### 2.2 Testing the workflow

To illustrate that Panalyze’s PVG analysis approach is broadly applicable to all virus genomes, a range of test datasets were analysed (Table 1). We used a computer cluster with Ubuntu v24.0 running on SLURM with 12 threads allocated per job, where each node had a Dual AMD EPYC 7763, 64 cores with 2 threads per core, 2.45 GHz processor and 2 GB memory. Firstly, we applied it to a set of 121 lumpy skin disease virus (LSDV) genomes, repre-senting large DNA viruses (taking 114 CPU hours, 78 minutes to complete). Secondly, we evaluated Panalyze’s performance on 15 porcine respiratory coronavirus (PRCV) genomes, representing small ssRNA viruses (6.7 CPU hours, 6.8 minutes). We repeated this on (ssRNA) 18 FMDV serotype C genomes (2.0 CPU hours, 2.6 minutes), and 441 FMDV serotype O genomes (353 CPU hours, 518 minutes). Thirdly, we evaluated its effectiveness in interpreting a segmented ssRNA virus, Rift Valley fever virus (RVFV), whose genome has three segments (S in 15 CPU hours/7.1 minutes, M in 18 CPU hours/10.3 minutes, L in 593 CPU hours, 553 minutes). Fourthly, we demonstrate the scalability of Panalyze by applying it to 2358 mpox genomes (23 CPU hours, 249 minutes).

**Table 1:** Properties of selected virus PVGs.

Virus	Region	Samples	Scaffold (bp)	Length (bp)	Nodes	Edges	Openness
FMDV A	Genome	142	8028	15 793	13 102	21 235	0.81
FMDV C	Genome	18	8148	10 779	5959	8359	1.14
FMDV O	Genome	441	8178	16 879	14 064	22 993	0.99
GTPV	Genome	6	150 350	163 590	7523	10 235	1.92
LSDV	Genome	121	150 524	156 966	7203	9827	1.88
LSDV 5’ end	7.5 Kb	132	7500	8954	790	1037	1.80
LSDV 3’ end	5 Kb	132	5001	7198	443	572	1.64
MPOX	Genome	2358	197 051	280 783	32 387	46 230	0.60
PRCV	Genome	15	27 660	32 086	7559	10 233	1.13
RVFV	S segment	414	1690	2739	1738	2495	0.97
RVFV	M segment	302	3885	6182	3639	5091	0.93
RVFV	L segment	306	6399	9092	5528	7677	0.99
SPPV	Genome	31	149 834	151 742	3136	4354	1.04
FMDV A	Genome	142	8028	15 793	13 102	21 235	0.81
FMDV C	Genome	18	8148	10 779	5959	8359	1.14
FMDV O	Genome	441	8178	16 879	14 064	22 993	0.99

The Scaffold refers to the average scaffold size, which was from GFAstats such that the total length refers to the sum of the sequence lengths of the nodes in the PVG from ODGI. FMDV A stands for FMDV serotype A. FMDV C stands for FMDV serotype C. FMDV O stands for FMDV serotype O. Openness was estimated using the alpha values from Panacus.

We repeated the analyses of the same sets of viral genomes using the nf-core/pangenome pipeline (Heumos *et al.* 2024) for comparison with Panalyze using the same sets of viral genomes, though the nf-core/pangenome pipeline required at least 12 threads. Metrics are extracted for the pruned PVGs to ensure con-sistency of comparisons.

Panalyze and the nf-core/pangenome pipeline provided idential results and the nf-core/pangenome pipe-line was faster overall. However, the RVFV segments S and M were too small to be assessed by nf-core/pangenome. In addition, Panalyze performs a wide range of PVG analysis and visualisa-tions absent in nf-core/pangenome.

To run Panalyze on laptop environments, we created two in-stances of virtual machines on a linux laptop having 64 GB RAM, and 32 cores. The high-resource instance had four cores with 8 GB RAM and the low-resource one had a single core with 4 GB RAM. We used a test dataset of 800 bp from the RVFV S segment for eight genomes running on a single thread. We were also able to run our pipeline on a windows virtual machine with eight cores and 8 GB of RAM.

## 3 Results

We created PVGs for large DNA (LSDV, mpox, GTPV, SPPV), small ssRNA (PRCV, FMDV) and segmented RNA (RVFV) viruses using Panalyze to illustrate its broad applicability to different virus types, as well as levels of diversity, sequence sizes and sample sizes (Table 1). The varied relative rate of nodes and edges compared to the sample sizes and average genome lengths illustrate how different the levels of sequence variability were. To illustrate the unusual patterns of variation in certain viral genomes, we extracted a portion of the LSDV PVG at which there are 15 mutations spanning 2 genes (Fig. 1E). Phylogenetic reconstruction shows divergent haplotypes: selecting a single representative reference sequence for this region would bias downstream analyses.

We modelled the rate of discovery of new mutations as a function of the number of samples added to the PVGs, estimated with Panacus calculation of alpha. We observed that RVFV’s segments had a slightly open PVG based on the alpha values from Panacus, whereas PRCV and the FMDV serotypes had slightly closed ones, which contrasted with the closed LSDV PVG and the open mpox PVG (Table 1). Similar values were observed with Panacus. Our estimate of gamma for LSDV’s PVG (Panacus 0.04) was comparable to a previous estimate of 0.05 derived from a gene-based pangenome (Xie *et al.* 2024).

## 4 Discussion

We present Panalyze: a portable pipeline for virus PVG construction, analysis and annotation written in domain-specific language (DSL) 2. It runs in NextFlow (Di Tommaso *et al.* 2017) using Docker (Merkel 2014) containers, and allows Nextflow’s process management to improve thread allocation and accelerate task completion. The selection of tools to run can be specified using a configuration file. This automation facilitates deeper engagement with complex datasets and it can analyse genomic regions of any size. It can run on any number of threads (from one to many), allowing it to be run locally on a laptop as well as on an HPC server. Panalyze can be applied to diverse viruses: large DNA, small ssRNA, and segmented ones.

Although there are emerging tools for PVG construction, analysis and annotation for smaller genomes, no tool has yet bridged these nor automated them in Nextflow. Panalyze addresses this in a user-oriented reproducible manner. We hope that Panalyze can help the virus research community progress from using linear reference genomes to more accurate PVGs generated from more representative datasets. Moreover, we expect that superior PVG methods will underpin better novel or recombinant sample characterisation as well as routine PVG analysis and mutation detection. Consequently, Panalyze supports virus evolutionary and epidemiological genomics.

## Supplementary Material

vbag071_Supplementary_Data

## Data Availability

The data underlying this article are available on GitHub at https://github.com/downingtim/Panalyze.

## References

[vbag071-B1] Alser M , Rotman J, Deshpande D et al Technology dictates algorithms: recent developments in read alignment. Genome Biol 2021;22:249. 10.1186/s13059-021-02443-7 34446078 PMC8390189

[vbag071-B2] Baaijens JA , Aabidine AZE, Rivals E et al De novo assembly of viral quasispecies using overlap graphs. Genome Res 2017;27:835–48. 10.1101/gr.215038.116 28396522 PMC5411778

[vbag071-B3] Beyer W , Novak AM, Hickey G et al Sequence tube maps: making graph genomes intuitive to commuters. Bioinformatics 2019;35:5318–20. 10.1093/bioinformatics/btz597 31368484 PMC6954646

[vbag071-B4] Boehm E , Kronig I, Neher RA, et al Novel SARS-CoV-2 variants: the pandemics within the pandemic. Clin Microbiol Infect 2021;27:1109–17. 10.1016/j.cmi.2021.05.022 34015535 PMC8127517

[vbag071-B5] Bradley P , den Bakker HC, Rocha EPC et al Ultrafast search of all deposited bacterial and viral genomic data. Nat Biotechnol 2019;37:152–9. 10.1038/s41587-018-0010-1 30718882 PMC6420049

[vbag071-B6] Chen NC , Paulin LF, Sedlazeck FJ et al Improved sequence mapping using a complete reference genome and lift-over. Nat Methods 2024;21:41–9. 10.1038/s41592-023-02069-6 38036856 PMC11610747

[vbag071-B7] Chen NC , Solomon B, Mun T et al Reference flow: reducing reference bias using multiple population genomes. Genome Biol 2021;22:8. 10.1186/s13059-020-02229-3 33397413 PMC7780692

[vbag071-B8] Colquhoun RM , Hall MB, Lima L et al Pandora: nucleotide-resolution bacterial pan-genomics with reference graphs. Genome Biol 2021;22:267. 10.1186/s13059-021-02473-1 34521456 PMC8442373

[vbag071-B9] Darriba D , Posada D, Kozlov AM et al ModelTest-NG: a new and scalable tool for the selection of DNA and protein evolutionary models. Mol Biol Evol 2020;37:291–4. 10.1093/molbev/msz189 31432070 PMC6984357

[vbag071-B10] Di Tommaso P , Chatzou M, Floden EW et al Nextflow enables reproducible computational workflows. Nat Biotechnol 2017;35:316–9. 10.1038/nbt.3820 28398311

[vbag071-B11] Downing T. Approaches to studying virus pangenome variation graphs. Genom Proteom Bioinform 2026;qzag003. 10.1093/gpbjnl/qzag003 PMC13433262 41632591

[vbag071-B12] Duchen D , Clipman S, Vergara C, Thio CL, Thomas DL, Duggal P, Wojcik GL. A hepatitis B virus (HBV) sequence variation graph improves sequence alignment and sample-specific consensus sequence construction for genetic analysis of HBV. PLoS One 2024;19:e0301069. 10.1371/journal.pone.0301069 38669259 PMC11051683

[vbag071-B13] Eddelbuettel D , Balamuta JB. Extending R with C++: a brief introduction to Rcpp; 2018. 10.1080/00031305.2017.1375990.

[vbag071-B14] Eddelbuettel D , Sanderson C. RcppArmadillo: accelerating R with high-performance C++ linear algebra. Comput Stat Data Anal 2014;71:1054–63. 10.1016/j.csda.2013.02.005

[vbag071-B15] Ewels P , Magnusson M, Lundin S et al MultiQC: summarize analysis results for multiple tools and samples in a single report. Bioinformatics 2016;32:3047–8. 10.1093/bioinformatics/btw354 27312411 PMC5039924

[vbag071-B52] Fischer C. GFAUTIL. 2021. https://crates.io/crates/gfautil

[vbag071-B16] Formenti G , Abueg L, Brajuka A et al Gfastats: conversion, evaluation and manipulation of genome sequences using assembly graphs. Bioinformatics 2022;38:4214–6. 10.1093/bioinformatics/btac460 35799367 PMC9438950

[vbag071-B17] Garrison E , Guarracino A. Unbiased pangenome graphs. Bioinformatics 2023;39:btac743. 10.1093/bioinformatics/btac743 36448683 PMC9805579

[vbag071-B18] Garrison E , Guarracino A, Heumos S et al Building pangenome graphs. Nat Methods 2024;21:2008–12. 10.1038/s41592-024-02430-3 39433878

[vbag071-B19] Garrison E , Sirén J, Novak AM et al Variation graph toolkit improves read mapping by representing genetic variation in the reference. Nat Biotechnol 2018;36:875–9. 10.1038/nbt.4227 30125266 PMC6126949

[vbag071-B20] Guarracino A , Buonaiuto S, de Lima LG, et al Recombination between heterologous human acrocentric chromosomes. Nature 2023;617:335–43. 10.1038/s41586-023-05976-y 37165241 PMC10172130

[vbag071-B21] Guarracino A , Heumos S, Nahnsen S et al ODGI: understanding pangenome graphs. Bioinformatics 2022;38:3319–26. 10.1093/bioinformatics/btac308 35552372 PMC9237687

[vbag071-B22] Guarracino A , Mwaniki N, Marco-Sola S et al wfmash: whole-chromosome pairwise alignment using the hierarchical wavefront algorithm; 2021. https://github.com/ekg/wfmash.

[vbag071-B23] Haga IR , Shih BB, Tore G et al Sequencing and analysis of lumpy skin disease virus whole genomes reveals a new viral subgroup in West and Central Africa. Viruses 2024;16:557. 10.3390/v16040557 38675899 PMC11053774

[vbag071-B24] Hein J. A new method that simultaneously aligns and reconstructs ancestral sequences for any number of homologous sequences, when the phylogeny is given. Mol Biol Evol 1989;6:649–68. 10.1093/oxfordjournals.molbev.a040577 2488477

[vbag071-B25] Heumos S , Heuer ML, Hanssen F, Heumos L, Guarracino A, Heringer P, Ehmele P, Prins P, Garrison E, Nahnsen S. Cluster-efficient pangenome graph construction with nf-core/pangenome. Bioinformatics 2024;40:btae609. 10.1093/bioinformatics/btae609 39400346 PMC11568064

[vbag071-B26] Hickey G , Heller D, Monlong J et al Genotyping structural variants in pangenome graphs using the vg toolkit. Genome Biol 2020;21:35. 10.1186/s13059-020-1941-7 32051000 PMC7017486

[vbag071-B27] Katoh K , Standley DM. MAFFT: iterative refinement and additional methods. Methods Mol Biol 2014;1079:131–46. 10.1007/978-1-62703-646-7_8 24170399

[vbag071-B28] Kozlov AM , Darriba D, Flouri T et al RAxML-NG: a fast, scalable and user-friendly tool for maximum likelihood phylogenetic inference. Bioinformatics 2019;35:4453–5. 10.1093/bioinformatics/btz305 31070718 PMC6821337

[vbag071-B29] Li H , Handsaker B, Wysoker A, et al The sequence alignment/map format and SAMtools. Bioinformatics 2009;25:2078–9. 10.1093/bioinformatics/btp352 19505943 PMC2723002

[vbag071-B30] Lischer HEL , Shimizu KK. Reference-guided de novo assembly approach improves genome reconstruction for related species. BMC Bioinf 2017;18:474. 10.1186/s12859-017-1911-6 PMC5681816 29126390

[vbag071-B31] Manni M , Berkeley MR, Seppey M et al BUSCO update: novel and streamlined workflows along with broader and deeper phylogenetic coverage for scoring of eukaryotic, prokaryotic, and viral genomes. Mol Biol Evol 2021;38:4647–54. 34320186 10.1093/molbev/msab199 PMC8476166

[vbag071-B32] Merkel D. Docker: lightweight linux containers for consistent development and deployment. Linux J 2014;2:2 https://10.5555/2600239.2600241

[vbag071-B33] Moshiri N , Fisch KM, Birmingham A et al The ViReflow pipeline enables user friendly large scale viral consensus genome reconstruction. Sci Rep 2022;12:5077. 10.1038/s41598-022-09035-w 35332213 PMC8943356

[vbag071-B34] Ondov BD , Treangen TJ, Melsted P et al Mash: fast genome and metagenome distance estimation using MinHash. Genome Biol 2016;17:132. 10.1186/s13059-016-0997-x 27323842 PMC4915045

[vbag071-B35] Paradis E , Schliep K. Ape 5.0: an environment for modern phylogenetics and evolutionary analyses in R. Bioinformatics 2019;35:526–8. 10.1093/bioinformatics/bty633 30016406

[vbag071-B36] Parmigiani L , Garrison E, Stoye J, Marschall T, Doerr D. Panacus: fast and exact pangenome growth and core size estimation. Bioinformatics. 2024a;40:btae720. 10.1093/bioinformatics/btae720 39626271 PMC11665632

[vbag071-B37] Parmigiani L , Wittler R, Stoye J. Revisiting pangenome openness with k-mers. Peer Commun J 2024b;4:e47. doi: 10.24072/pcjournal.415

[vbag071-B38] Paten B , Eizenga JM, Rosen YM et al Superbubbles, ultrabubbles, and cacti. J Comput Biol. 2018;25:649–63. 10.1089/cmb.2017.0251 29461862 PMC6067107

[vbag071-B8709187] Paten B, , Novak AM, , Eizenga JM, Garrison E. Genome graphs and the evolution of genome inference. Genome Res 2017;27:665–76. 10.1101/gr.214155.116 28360232 PMC5411762

[vbag071-B39] R Core Team. R: A Language and Environment for Statistical Computing. Vienna, Austria: R Foundation for Statistical Computing, 2025. https://www.R-project.org/

[vbag071-B40] Rakocevic G , Semenyuk V, Lee W-P et al Fast and accurate genomic analyses using genome graphs. Nat Genet 2019;51:354–62. 30643257 10.1038/s41588-018-0316-4

[vbag071-B41] Revll LJ. Phytools 2.0: an updated R ecosystem for phylogenetic comparative methods (and other things). PeerJ 2024;12:e16505. 10.7717/peerj.16505 38192598 PMC10773453

[vbag071-B42] Sayers EW , Beck J, Bolton EE, et al Database resources of the national center for biotechnology information. Nucleic Acids Res 2021;49:D10–17. 10.1093/nar/gkaa892 33095870 PMC7778943

[vbag071-B43] Schliep KP. Phangorn: phylogenetic analysis in R. Bioinformatics 2011;27:592–3. 10.1093/bioinformatics/btq706 21169378 PMC3035803

[vbag071-B44] Seemann T. Prokka: rapid prokaryotic genome annotation. Bioinformatics 2014;30:2068–9. 10.1093/bioinformatics/btu153 24642063

[vbag071-B45] Valiente-Mullor C , Beamud B, Ansari I, et al One is not enough: on the effects of reference genome for the mapping and subsequent analyses of short-reads. PLoS Comput Biol 2021;17:e1008678. 10.1371/journal.pcbi.1008678 33503026 PMC7870062

[vbag071-B46] Wang L-G , Lam TT-Y, Xu S et al Treeio: an R package for phylogenetic tree input and output with richly annotated and associated data. Mol Biol Evol 2020;37:599–603. 10.1093/molbev/msz240 31633786 PMC6993851

[vbag071-B47] Wick RR , Schultz MB, Zobel J et al Bandage: interactive visualization of de novo genome assemblies. Bioinformatics 2015;31:3350–2. 10.1093/bioinformatics/btv383 26099265 PMC4595904

[vbag071-B48] Wickham H. ggplot2: Elegant Graphics for Data Analysis. New York: Springer-Verlag; 2016. https://ggplot2.tidyverse.org

[vbag071-B49] Wright C , Tennakoon C, Lasecka-Dykes L et al Using pangenome variation graphs to improve mutation detection in a large DNA virus. BioRxiv 2025. doi: 10.1101/2025.11.26.690900 PMC13189362 42160133

[vbag071-B50] Xie S , Cui L, Liao Z et al Genomic analysis of lumpy skin disease virus asian variants and evaluation of its cellular tropism. NPJ Vaccines 2024;9:65. 10.1038/s41541-024-00846-8 38514651 PMC10957905

[vbag071-B51] Yu G , Smith D, Zhu H et al Ggtree: an R package for visualization and annotation of phylogenetic trees with their covariates and other associated data. Methods Ecol Evol 2017;8:28–36. 10.1111/2041-210X.12628

